# Investigation of the Seroprevalence of *Lentivirus ovivismae* and *Lentivirus capartenc* (Formerly Known as Maedi‐Visna and Caprine Arthritis‐Encephalitis Virus) Infections in Sheep and Goats in the Yozgat Region

**DOI:** 10.1002/vms3.70911

**Published:** 2026-03-31

**Authors:** Emre Sayar, Secil Sevinc Temizkan, Akın Kırbaş, Güvenç Gökalp

**Affiliations:** ^1^ Department of Internal Medicine Faculty of Veterinary Medicine Yozgat Bozok University Yozgat Türkiye; ^2^ Department of Virology Faculty of Veterinary Medicine Yozgat Bozok University Yozgat Türkiye; ^3^ Department of Internal Medicine Faculty of Veterinary Medicine Atatürk University Erzurum Türkiye; ^4^ Department of Internal Medicine Faculty of Veterinary Medicine Yozgat Bozok University Yozgat Türkiye

**Keywords:** caprine arthritis, *Lentivirus capartenc*, *Lentivirus ovivismae*, maedi‐visna, seroprevalence

## Abstract

**Background:**

*Lentivirus ovivismae* and *Lentivirus capartenc* (formerly maedi‐visna and caprine arthritis‐encephalitis virus) are highly contagious, chronic viral infections that cause significant economic losses in small ruminant farming. This study aimed to determine the current seroprevalence of these infections in the Yozgat region, where sheep and goat farming is widespread and clinical signs are frequently observed.

**Materials and Methods:**

Blood samples were collected from 160 sheep and 160 goats in districts with high small ruminant populations, including Sorgun, Sarıkaya, Saraykent, Boğazlıyan, Şefaatli and Akdağmadeni. The samples were analysed using *Lentivirus ovivismae* and *Lentivirus capartenc* enzyme‐linked immunosorbent assay (ELISA) tests to detect the presence of antibodies.

**Results:**

Out of the 320 animals sampled, 10 sheep (6.25%) tested positive for *Lentivirus ovivismae* and 28 goats (17.5%) tested positive for *Lentivirus capartenc*. These results demonstrate a notable level of viral circulation within the livestock population of the Yozgat Province.

**Conclusion:**

The findings indicate that both infections are prevalent in the region, posing a threat to productivity. This study serves as a baseline for informing local veterinarians and farmers about preventive measures. Based on these data, further comprehensive studies and control strategies are planned for the region.

## Graphical abstract

This study conducted in the Yozgat region determined a prevalence of 6.35% for maedi‐visna in sheep and 17.5% for caprine arthritis encephalitis in goats, providing critical data for small ruminant health and disease management in the area.

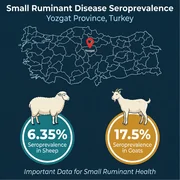

## 1 Introduction

According to the revised taxonomic classification published by the International Committee on Taxonomy of Viruses (ICTV) in 2023, the etiological agent previously known as caprine arthritis‐encephalitis virus (CAEV) has been reclassified as *Lentivirus capartenc*, and the agent formerly referred to as maedi‐visna virus (MVV) is now named *Lentivirus ovivismae*. The term maedi refers to dyspnoea, while visna denotes shrinkage or weakness resulting from meningoencephalitis. Hence, *Lentivirus ovivismae*, previously known as MVV, manifests with a variety of clinical signs such as progressive loss of body condition, reduced mobility, increased respiratory rate, pneumonia, coughing, nasal discharge, muscle tremors, ataxia, partial or complete paralysis and blindness often culminating in death. Additionally, the virus may also cause mastitis and arthritis. *Lentivirus capartenc*, formerly known as CAEV, is associated with arthritis, encephalitis and pneumonia, and may also contribute to the development of mastitis (Highland 2017; Kalaycı et al. 2023). Although *Lentivirus ovivismae* is more commonly observed in sheep and *Lentivirus capartenc* in goats, both viruses are capable of infecting both species (Blacklaws 2012).

Due to their structural, genetic and pathogenic similarities, these viruses collectively known as small ruminant lentiviruses (SRLVs) are classified within the genus *Lentivirus*, a member of the Retroviridae family, subfamily Orthoretrovirinae (Coffin et al. 2021). Like other retroviruses, retroviral particles contain two copies of linear single‐stranded RNA, which are reverse transcribed into double‐stranded DNA (dsDNA) by the enzyme reverse transcriptase (RT). This newly synthesized dsDNA, referred to as the provirus, is subsequently integrated into the genome of the host cell. The viral nucleic acid encodes three major structural proteins: env, gag and pol. The pol gene, located centrally within the viral genome, encodes essential enzymes involved in replication and integration processes, including protease (PR), RT, dUTPase and integrase (Gomez‐Lucia et al. 2018).

These viruses, described by organizations such as the World Organisation for Animal Health (WOAH/OIE) and the Food and Agriculture Organization (FAO) as ‘widespread yet often neglected’ infections, are known to persist globally and have been the subject of numerous studies conducted in various countries (Cecco et al. 2022; Wu et al. 2022; Peterson et al. 2022; Murphy et al. 2021). In Türkiye as well, numerous studies have been carried out to date (Kalaycı et al. 2023; Burgu 1990; Timurkan et al. 2024; Albayrak et al. 2012; Ameen and Karapınar 2018; Arık et al. 2015; Çelik et al. 2018; Doğan et al. 2021; Eroksuz et al. 2022; Gezer et al. 2021; Girgin et al. 1987; Gürçay and Parmaksız 2013; Taşkaya and Kale 2020; Karapınar et al. 2016; Muz et al. 2013; Tan and Alkan 2002; Ün et al. 2018; Yilmaz et al. 2002; Yavru et al. 2006). Prevalence data play a crucial role in the control of these viruses, and such studies should be continuously maintained.

Among the infections affecting sheep and goats, lentiviral infections are distinct from other viral diseases due to the absence of vaccines, their slow progression and their lifelong persistence within the host (de Andrés et al. 2005). The early and accurate diagnosis of these infections is the cornerstone of all control and eradication efforts (Kalogianni et al. 2021). For the implementation of effective control and eradication programs, up‐to‐date epidemiological data from the relevant region are essential. This study aims to provide current data on the status of *Lentivirus ovivismae* and *Lentivirus capartenc* infections in the central and surrounding districts of Yozgat Province, where sheep and goat farming is widespread. The findings of this research are expected to serve as a foundation for future advanced studies.

## 2 Materials and Methods

In this study, the seroprevalence of *Lentivirus ovivismae* and *Lentivirus capartenc* infections was determined in sheep and goat farms located in regions with a high small ruminant population in Yozgat (Sorgun, Sarıkaya, Saraykent, Boğazlıyan, Şefaatli and Akdağmadeni). The study samples were selected using a randomized method. The small ruminant farms from which the samples were taken were registered with the Yozgat Provincial Directorate of Food, Agriculture and Livestock and the Yozgat Sheep and Goat Breeders’ Association. Anamnesis information of the farm owners was recorded, and clinical examinations were performed to determine the age and number of animals on the farms.

**FIGURE 1:**
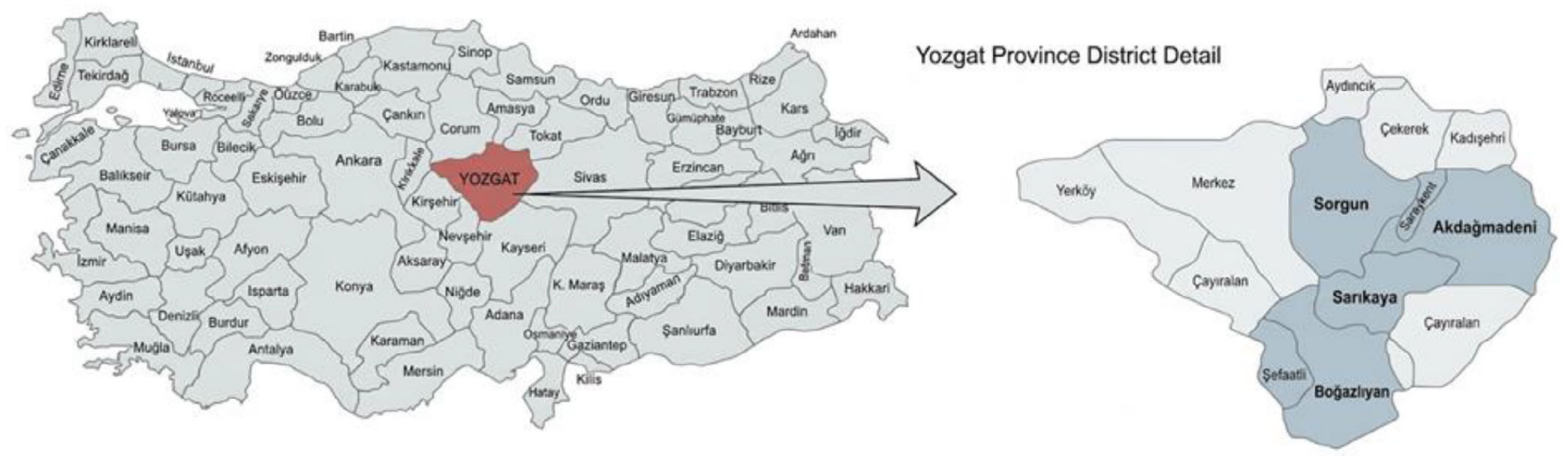
Map of the study area.

The infectious diseases studied (*Lentivirus ovivismae* and *Lentivirus capartenc*) are persistent infections that progress slowly and have an incubation period of 1–2 years. For this reason, samples were taken from lactating female (sheep and goats) and male animals (rams and bucks) aged 2 years and older, regardless of the time of year. As *Lentivirus ovivismae* and *Lentivirus capartenc* cause infections specific to sheep and goats, and due to the high population of Akkaraman sheep and hair goats in the specified region, these breeds were used in the study (Figure 1).

In order to determine the seroprevalence of SRLV, the sampling was carried out using a multi‐stage sampling method based on the population of 304,500 sheep and 54,100 goats in the Yozgat region in 2021, with a 95% confidence interval (CI), an estimated prevalence of 50% and a ±5% margin of error. Accordingly, a total of 320 animals were included in the project: 160 sheep/rams and 160 goats/bucks. General examinations were performed, and blood samples were collected in 5 mL yellow‐capped serum tubes. To obtain serum from the collected blood samples, centrifugation was performed at 3000 rpm for 10 min (Universal 320 R Hettich Zentrifugen). The obtained sera were transferred into 0.5 mL portions in Eppendorf tubes and stored at −20°C until enzyme‐linked immunosorbent assay (ELISA) tests were conducted. The sera were analysed using commercial ELISA antibody test kits specific for *Lentivirus ovivismae* (Sheep Maedi Visna Virus ELISA Kit, Cat No: ED0006Sh) and *Lentivirus capartenc* (Goat Caprine Arthritis Encephalitis Virus ELISA Kit, Cat No: ED0008GO), in accordance with the procedures provided in the kits.

### 2.1 Statistical Analysis

The study determined the total number of animals to be sampled in Yozgat city centre and its districts based on the registered small ruminant population (358,600) obtained from the Provincial Directorate of Agriculture. A power analysis was conducted using the G*Power 3.1 statistical program, and with an effect size of G: 0.10, α: 0.05 and a test power of 0.80, the minimum required sample size was determined to be 199. To enhance the reliability of the study and the frequency of seroprevalence detection, a total of 320 samples were collected, including 160 sheep and 160 goats.

### 2.2 Findings

According to the indirect ELISA results performed on the sera obtained from blood samples collected from six districts of Yozgat (Akdağmadeni, Boğazlıyan, Saraykent, Sarıkaya, Sorgun and Şefaatli) the data obtained are presented in Table 1.

**TABLE 1:** Seroprevalence of *Lentivirus ovivismae* and *Lentivirus capartenc* in sheep and goats across different districts of Yozgat Province.

Species	District	Sample size (*n*)	Positive (*n*)	Seroprevalence (%)	95% confidence interval (CI)
**Sheep**	Akdağmadeni	26	0	0.00	0.00–13.23
(*L. ovivismae*)	Boğazlıyan	27	1	3.70	0.09–19.04
	Saraykent	27	2	7.41	0.91–24.29
	Sarıkaya	27	3	11.11	2.35–29.16
	Sorgun	26	2	7.69	0.95–25.13
	Şefaatli	27	2	7.41	0.91–24.29
	**Total (sheep)**	**160**	**10**	**6.25**	**3.05–11.18**
**Goats**	Akdağmadeni	26	3	11.54	2.45–30.15
(*L. capartenc*)	Boğazlıyan	27	4	14.81	4.19–33.73
	Saraykent	27	3	11.11	2.35–29.16
	Sarıkaya	27	10	37.04	19.40–57.63
	Sorgun	26	5	19.23	6.55–39.36
	Şefaatli	27	3	11.11	2.35–29.16
	**Total (goat)**	**160**	**28**	**17.50**	**11.96–24.28**

As seen in Table 1, the prevalence of *Lentivirus ovivismae* infection was determined to be 6.25%, whereas the prevalence of *Lentivirus capartenc* was found to be 17.5%. Although no clinical abnormalities were observed in any of the farms in the specified districts, the infections were detected serologically.

## 3 Discussion

In this study, the seroprevalence of *Lentivirus ovivismae* and *Lentivirus capartenc* infections in sheep and goats in Yozgat Province was determined. Alibaşoğlu and Arda (1975) stated that *Lentivirus ovivismae*, initially identified in sheep slaughtered in abattoirs in Turkey based on pathological findings, was later detected in both private and public enterprises across various regions in subsequent studies (Alibaşoğlu and Arda 1975). Girgin et al. (1987) reported the disease in two imported rams in 1984 based on clinical and pathological findings and observed an increasing number of studies in the following years (Girgin et al. 1987).

Similar to our study, it has been reported that the prevalence of *Lentivirus ovivismae* and *Lentivirus capartenc* varies across different provinces. A study conducted in Erzurum Province reported a seropositivity rate of 15.12% (Timurkan et al. 2024). In another study carried out in the Aegean Region, the seropositivity rate was found to be 24.42% in sheep and 6.55% in goats (Kalaycı et al. 2023). In a study from Yalova Province, based on *Lentivirus ovivismae* PCR and ELISA test results, positivity rates in sheep were 5.62% (13/231) and 5.19% (12/231), respectively (Sait and Ince 2022). In a study conducted in the Eastern Mediterranean Region where both ELISA and PCR were used, SRLVs were detected as positive at rates of 9.62% in sheep, 9.32% in goats and 9.43% in all animals (Doğan et al. 2021). A seropositivity rate of 16% for *Lentivirus ovivismae* was found in Kars Province (Gezer et al. 2021). In Burdur Province, a seropositivity rate of 1.6% for *Lentivirus capartenc* was reported (Taşkaya and Kale 2020). In a study focused solely on *Lentivirus ovivismae* conducted in northern Turkey, a seropositivity rate of 23.5% was identified (Albayrak et al. 2012). Another study carried out in and around Van Province found a *Lentivirus ovivismae* seropositivity rate of 10.5% (Ameen and Karapınar 2018). In a different study conducted in the Siirt region, both viruses were found to be seronegative in 465 animals (Çelik et al. 2018). In Şanlıurfa, the seroprevalence of *Lentivirus ovivismae* infection was determined to be 5.29% (Ün et al. 2018). In another study involving blood and milk samples collected from various regions of Turkey (province names not specified), *Lentivirus capartenc* was investigated using ELISA and PCR. According to the study results, 8.5% of blood samples tested positive by ELISA, while 4.9% of milk samples were positive. PCR results showed positivity rates of 20% in blood samples and 50% in milk samples (Karapınar et al. 2016). In Afyonkarahisar and its surroundings, a *Lentivirus ovivismae* seropositivity rate of 5.7% was reported (Arık et al. 2015). In Şanlıurfa, another study found a 21.1% seropositivity rate for *Lentivirus ovivismae* (Gürçay and Parmaksız 2013). Additionally, in a study sampling different regions of Turkey, the prevalence of *Lentivirus ovivismae* was determined to be 67.8% using ELISA and/or LTR‐PCR (Muz et al. 2013). Another study reported a 26.7% prevalence of *Lentivirus ovivismae* (Tan and Alkan 2002).

Similar to our study, another study conducted in the Yozgat and Konya regions used 190 goats and employed both agar gel immunodiffusion (AGID) and ELISA tests. As a result, ELISA was reported to be more reliable. According to the test results, *Lentivirus capartenc* (CAEV) seropositivity rates of 1.57% and 3.68% were detected using AGID and ELISA tests, respectively (Yavru et al. 2006). In the study mentioned above, the animals were kept in two different state farms. Since these are state farms, it is assumed that a more organized breeding program was implemented, which may explain why the seropositivity rate for CAEV infection was lower compared to our study. In contrast, the samples in our study were mostly collected from traditional farms, which likely contributed to the higher CAEV prevalence.

The positivity rates observed in our study differ from those reported in other studies. We believe that these differences are shaped by the small ruminant population in our region, as well as variations in season, breed and the intensity of animal movements.

Eradication of MVV was first accomplished in Iceland, before the cause was known or serologic tests were established, by an extensive slaughter program in which diseased and exposed sheep were killed and buried (Cutlip and Lehmkuhl 1986). In the second program, female lambs were separated from their dam just before birth without receiving colostrum. No *Lentivirus ovivismae* antibodies were detected in this flock. Although the first program was highly practical, the second program was reported to be more effective as it led to the earlier development of an MVV seronegative flock. Due to the nature of the humoral response, a period of more than 4 years is required to completely eradicate MVV from any herd.

The historical eradication programs in Iceland serve as a fundamental model for the control of SRLVs. The Icelandic experience demonstrated that even before the full characterization of the viruses, rigorous culling of seropositive animals and controlled colostrum management could successfully break the transmission cycle (Cutlip and Lehmkuhl 1986).

In light of the seropositivity rates identified in our study (6.25% in sheep and 17.5% in goats), implementing similar control strategies in the Yozgat Region is of critical importance. Specifically, for the farms where positivity was detected, we recommend a management protocol based on the isolation of seropositive animals from the flock. Furthermore, to prevent vertical transmission, newborn lambs and kids should be separated from seropositive dams immediately after birth and reared using colostrum from seronegative sources or pasteurized milk. Such bio‐security measures, adapted from successful global examples, are essential for reducing the viral load and ensuring the long‐term sustainability of small ruminant farming in the region.

## 4 Conclusion

As a result of the study, the prevalence of *Lentivirus ovivismae* infection was determined to be 6.25%, while the prevalence of *Lentivirus capartenc* infection was found to be 17.5%. Based on the findings obtained from this study, it is considered beneficial for disease control and eradication efforts to continuously monitor *Lentivirus ovivismae* and *Lentivirus capartenc* infections, conduct periodic seroepidemiological studies, perform on‐site pre‐tests during importation and select animals accordingly. In particular, providing consultancy support to breeding farms with high genetic value and focusing on awareness‐raising activities for farmers are of great importance. The status of positivity in the indicated farms will be shared with the farm owners to ensure they are informed about the disease. Through this approach, it is aimed to at least partially prevent these infections that cause abortions. According to these findings and results, it is thought that this study will serve as a model for future similar or more detailed studies and help fill the gap in the literature regarding Yozgat.

## Author Contributions

**Emre Sayar**: conceptualization, data curation, funding acquisition, investigation, methodology, data validation, formal analysis, project administration, supervision, review and editing of the manuscript, visualization. **Secil Sevinc Temizkan**: conceptualization, data validation, formal analysis, project administration, supervision. **Akın Kırbaş**: investigation, review and editing of the manuscript, visualization. **Güvenç Gökalp**: conceptualization, drafting the manuscript, formal analysis.

## Ethics Statement

The study was approved by the Erciyes University Local Ethics Committee for Animal Experiments with the decision numbered 22/043 on 02.02.2022.

## Conflicts of Interest

The authors declare no conflicts of interest.

## Data Availability

The datasets generated during and/or analysed during the current study are available from the corresponding author on reasonable request.
